# A quest for global psychology

**DOI:** 10.1007/s43638-022-00044-0

**Published:** 2022-11-23

**Authors:** Luca Tateo, Giuseppina Marsico

**Affiliations:** 1 University of Oslo, Oslo, Norway; 2 Federal University of Bahia, Salvador da Bahia, Brazil; 3 University of Salerno, Fisciano, Italy

**Keywords:** Ecosystemic Psychology, Psychodiversity, Anthropophagic Manifesto, Decolonising, Cultural Psychology, Ökosystemische Psychologie, Psychodiversität, Anthropophagisches Manifest, Entkolonialisierung, Kulturelle Psychologie

## Abstract

In this article we discuss the decolonisation of psychology by constructing a project that is open to diversity and transdisciplinarity, rather than providing hyper-fragmented technical knowledge. In the iconic *Manifesto antropófago* (1928), the poet Oswald de Andrade (1890–1954) claimed the original and creative capability of Brazilian modernist culture to elaborate in original ways the European, Indio and African heritage. We discuss the anthropophagic metaphor to elaborate on human phenomena that take place in “arenas” where complementary and (often) opposing views are at stake; where the people make their own personal synthesis through coordinated “processes of creating, managing, demolishing and rebuilding” meanings about themselves and the world. Research cannot be reduced to a competition between views that strive to prevail and occupy academic niches. Instead, it should be aimed at being a collective effort of understanding through dialogue.

An innovative epistemology shall not reject any emerging idea because it belongs to a different “species” or “perspective”. It is not hegemonic, rather, it is open to the construction of knowledge through dialogue and complementarity of views. The idea of Anthropophagic Psychology rejects a “monological” epistemology and instead allows for the development of a *polyphonic psychology*, an arena, i.e. a local ecosystem, where the polyphony of perspectives can lead to a rich epistemic orchestration.

## Abstract (de)

In diesem Artikel erörtern wir die Entkolonialisierung der Psychologie mithilfe eines Projekts, das offen für Vielfalt und Transdisziplinarität ist, anstatt lediglich hyperfragmentiertes technisches Wissen zu liefern. In seinem ikonischen Manifesto antropófago (1928) behauptete der Dichter Oswald de Andrade (1890–1954) die ursprüngliche und kreative Fähigkeit der brasilianischen Kultur der Moderne, das europäische, indianische und afrikanische Erbe auf originelle Weise integriert zu verarbeiten. Wir diskutieren seine anthropophagische Metapher, um menschliche Phänomene in „Arenen“ zu untersuchen, in denen komplementäre und (oft) gegensätzliche Ansichten auf dem Spiel stehen; in denen die Menschen ihre persönliche Synthese durch koordinierte „Prozesse der Erzeugung, Verwaltung, Zerstörung und Wiederherstellung“ von Bedeutungen ihrer selbst und der Welt schaffen. Die Forschung darf nicht auf einen Wettkampf von Ansichten reduziert werden, die sich durchsetzen und akademische Nischen besetzen wollen. Stattdessen sollte sie auf eine kollektive Anstrengung des Verständnisses durch Dialog zielen.

Eine innovative Erkenntnistheorie darf keine neue Idee ablehnen, nur weil sie einer anderen „Spezies“ oder „Perspektive“ angehört. Sie ist nicht hegemonial, sondern offen für dialogische Konstruktion von Wissen auf Basis der Komplementarität von Ansichten. Die Idee der anthropophagen Psychologie lehnt eine „monologische“ Erkenntnistheorie ab und ermöglicht stattdessen die Entwicklung einer polyphonen Psychologie, einer Arena, d. h. eines lokalen Ökosystems, in dem die Vielstimmigkeit der Perspektiven zu einer reichhaltigen epistemischen Orchestrierung führen kann.

## Prologue

**Fig. 1:**
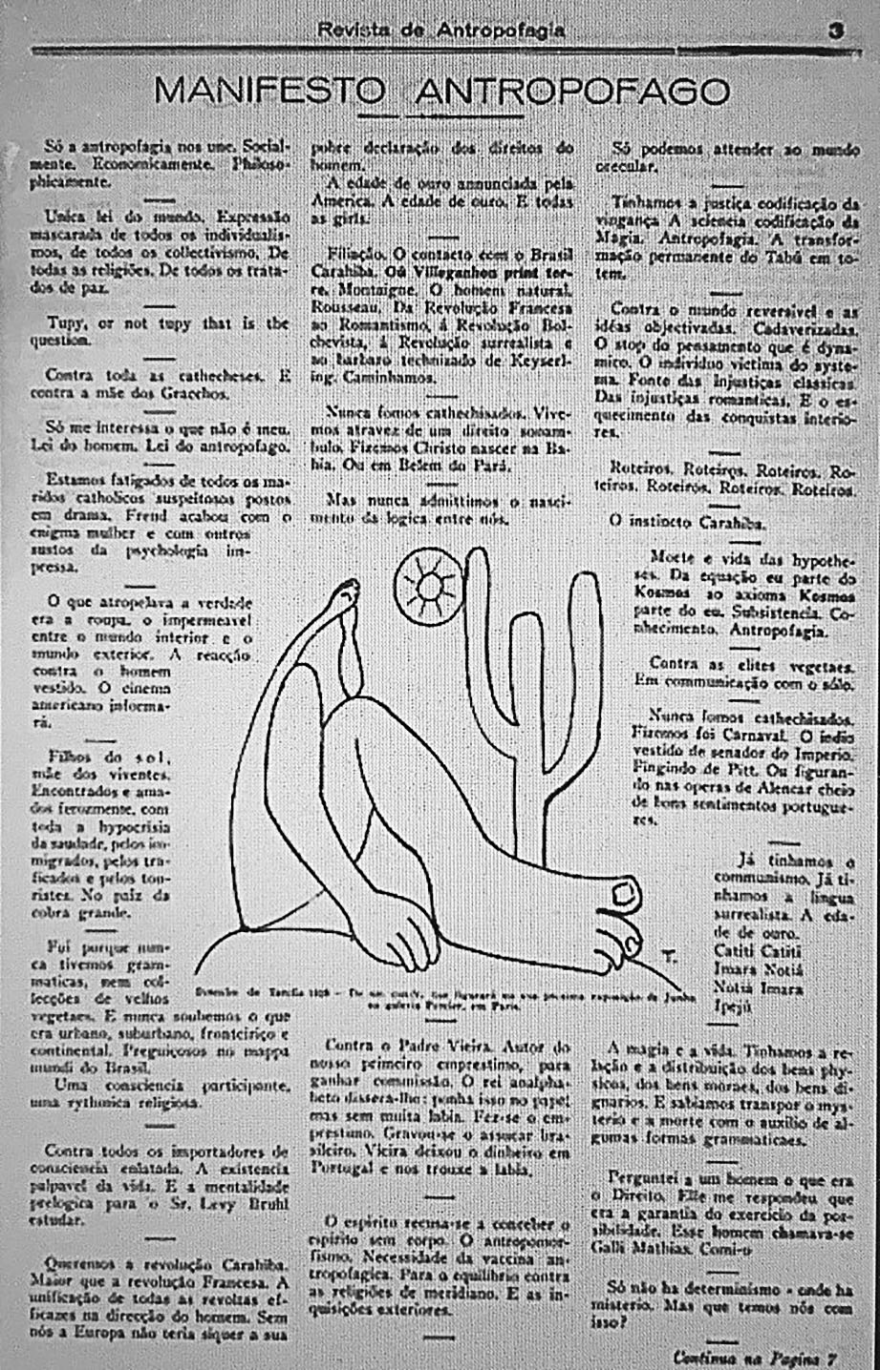
Original publication of “Manifesto Antropófago” in Revista de Antropofagia by Oswald de Andrade in 1928. The image at the centre is a contour line drawing by Brazilian artist Tarsila do Amaral from her 1928 painting “Abaporu”. (Creative Commons Licence, retrieved June 19, 2021 from URL https://commons.wikimedia.org/wiki/File:Manifesto_Antrop%C3%B3fago.jpg.)

In January 1928, Brazilian painter Tarsila do Amaral (1886–1973), presented her canvas *Abaporú *as a birthday gift to her husband, eminent modernist writer Oswald de Andrade (1890–1954). Through personal accounts of the couple, we learn that de Andrade was fascinated by the painting and proclaimed that he would create a movement around it. Using a dictionary of indigenous Brazilian languages, do Amaral and de Andrade named the painting *Abaporú*: which can be roughly translated as “man who eats”. A few months later, the painting became the cover of the *Manifesto Antropófago* (Anthropophagic Manifesto) (de Andrade 1928/ 1991), in which de Andrade argued that Brazil has a history of “cannibalising” other cultures, digesting them, and producing something entirely new (Fig. 1). The *Manifesto antropófago* continues to be an important reference for contemporary debate over hybridity and cultural dialogues. We have been inspired by the Manifesto to develop our argument about the future of psychological sciences in response to global challenges and the cultivation of *psychodiversity*.

## The challenges of psychological sciences

Although some psychologists have expressed their dissatisfaction with the individualistic, neopositivist and Western-centred approaches within the psychological sciences (Chakkarath 2010; Hwang 2015), others have found these approaches to be adequate and appropriate as part of the quest for a “scientific” psychology (Klempe 2020; Rieber 2013). Such a quest is indeed as old as the origins of social sciences as the knowledge of regulation (de Sousa Santos 2018). The idea of building a universal system of laws to describe, predict and regulate human behaviour would be the dream of every autocratic ruler. Making it “scientific” would ultimately legitimise such a dream under the garb of objective and “disinterested interest” (ibid.). The methodological fetishism and the supposed neutrality of research conceal ideological claims that support an order of power (ibid.). The monological myth of “evidence-based” psychological, social and educational sciences creates an “inherently asymmetrical” (ibid., p. 136) relation, in which those who are considered the object “cannot possibly appropriate the subject that knows it” (ibid., p. 136). By following the “proper” method, it is believed that:

“the truth about the object is obtained, and that it is the only relevant truth. It is an unwarranted belief since the methodology only provides us with those answers about the world that correspond to the questions asked by them in the first place, and such questions are only a fraction of those that could possibly be asked, indeed, we perceive the world as seemingly complete only because our questions about it are always very limited”. (ibid.)

Human phenomena, instead, take place in “arenas” where complementary and (often) opposing views are at stake, where the people make their own personal synthesis through coordinated processes of creating, managing, demolishing and rebuilding meanings about themselves and the world (Tateo and Marsico 2018). Thus, research cannot be a competition between opposing views that strive to prevail and occupy academic niches. Instead, it should be aimed at being a collective effort of understanding through dialogue. Nevertheless, every epistemology begins with a position. All knowledge originates from spatio-temporal coordinates and then either expands or shrinks the portion of visible universe one can grasp.

As authors, we therefore need to first position ourselves; to locate the origin coordinates of our perspective. We are two mid-career researchers, one woman and one man, both white and heterosexual, of South Italian origin but with a strong attitude towards international connections. With more than 20 years of research experience, we are currently working in an Italian university and a Norwegian university. We recognise that we are part of the privileged and elite psychologists that benefit from stable employment, ample salary, and adequate facilities to travel, publish and teach regularly. At times, we both feel uncomfortable with many aspects of the academic life, especially with hierarchical issues and with the corporative and neo-liberalist logic of present-time academia. Nevertheless, we are aware of our privilege as well as of the ethnocentric and limited perspectives that are dominating our field. We first became aware of this when we met mentors and colleagues from different contexts (i.e. Baltic, Latin America, Asia, Africa) who gave us the opportunity to encounter different ways of knowing, different values and different lifestyles. It was at this point that we realised that it was important to embrace a borderless and nomadic way of interpreting academic work.

Therefore, we feel the need to discuss the challenges that the psychological sciences will experience in the upcoming years. The current COVID-19 pandemic has revealed a number of contradictions that the capitalistic, neo-colonialist and patriarchal hegemonic way of life is cultivating. It has jeopardised the place of humans itself within the global ecosystem, and has uncovered all the limits of what was once believed to be the winning way of life and the culmination of human progress, after the main alternative models were dismissed during the late 1990s (Dege and Strasser 2022; Malm 2020). The pandemic is also challenging our epistemologies and presenting new global academic challenges. Psychology is not equipped with understanding and confronting such global crises that may continue to happen with increasing frequency in the future (Tateo and Marsico 2019). What one may call Western mainstream psychology is focused on the treatment of individual trauma, and assumes that an individual intervention based on behavioural, semiotic, emotional, cognitive or neurological changes (depending on the theoretical background) will restore the ability to be a fully functional person within the current social order. By acknowledging psychological discomfort and suffering only as an individual matter, psychology commits an act of injustice. If the person and the society do not match, and such a conflict causes suffering, it is the *person* who *must* change, not the *society*. This trauma-informed, North-Western perspective is then universalised, despite growing voices that claim that this is just one of the different indigenous psychologies (Gonzalez and Silva Guimarães 2020). The outcome is the transfer of a psychological monoculture, cultivated according to a capitalistic model, which is slowly killing *psychodiversity*, similar to the manner in which intensive monoculture capitalistic agriculture is destroying *biodiversity* and *anthropodiversity* (Shiva 1992, 2020; Pérez and Marsico 2021), increasing the likelihood of ecological disasters on a global scale. As journalist Jesse Singal has aptly stated, we look for “quick fix” psychology (Singal 2021) the same way we look for fresh mangos all year round across the world.

## Challenging *psychodiversity*

The majority of approaches in cross-cultural psychology are actually about intra-cultural psychology. Individual cultures are often treated as homogeneous, rigid compartments that are responsible for individual differences. To fulfil requirements of the variable-based design, cultures are assumed to be isolated from each other (Gamsakhurdia 2018), as if to say that there exists no interaction. However, we know that this is not the real way people relate to cultures (Gamsakhurdia 2020). The actual intercultural dynamic of the encounter between people of different backgrounds is seldom considered, including the moment of the encounter with the researcher. Similarly, the researcher’s gaze is *normalising*, that is, the gold standard is the perspective of the (Global North) researcher’s cultural and academic values.

As psychologists, we are unfairly unaware of the costs of cultivating a permanent psychological monoculture for human *psychodiversity*. “The basic assumption or prejudice” that informs psychological Northern global epistemology “is to consider diversity as superficial (appearance) and unity as a profound (underlying structure)” (de Sousa Santos 2018, p. 39). Following de Sousa Santos (2018), we refer to epistemologies of the North as a hegemonic, dominant and oppressive way of promoting a monological vision of the human being and its relationship with the environment. Such a vision has originated in the colonialist, capitalistic and patriarchal socio-economic order and it continues to persist through acts of violence, marginalising and silencing. The “North” is of course not a geographical place, rather a cognitive one (de Sousa Santos 2018). Monoculture, homogeneity and standardisation are sought by all oppressive systems, regardless of which continent they are located in. Cognitive monoculture is essential to a capitalistic economy the same way in which biological monoculture is essential to capitalistic agriculture.

There is a false sense of understanding of what unity represents. Psychological sciences confuse a monological discourse for an ecological one: no ecosystem is made of just one kind of organism. Systemic differentiation and polyphony are key characteristics of living wholes (Diriwachter and Valsiner 2011). Local, dynamic–systemic unity and hierarchically organised diversity are both complementary characteristics of any living ecosystem. Failing to incorporate this fundamental principle into the epistemology of psychology will be a deathblow to the future of the discipline and will render it incapable of facing future global challenges.

We aim to foster a discussion about the future of a global psychology that is able to cultivate *psychodiversity *(Tateo 2021), that is the manyfold forms of psychological organisation that develop different ecosystems. We would also like to inquire how we can promote “synthetic” and “syncretic” theorising in psychology (Tateo 2020), which refers to a form of theorising that acknowledges *psychodiversity* and produces generalisable models of psychic processes, instead of typological differences and similarities (Valsiner 1984).

## The history of the Anthropophagic Manifesto

The Brazilian modernist anthropophagic movement was created in the late 1920s by de Andrade and others, who published the *Revista de Antropofagia* (1928–1929) (Fig. 1).

In the 1920s, Brazil was a country experiencing growing urban industrialisation. However, it was mainly characterised as an exporter of coffee and other raw materials, as well as a country that was associated with a history of slavery, formally abolished only in 1888.

Brazil also imported the latest trends of European culture, such as futurism, dadaism, cubism, modern architecture and psychoanalysis, along with expensive cars, fashion and technological innovations (as well as the cheap labour of European immigrants) (Jáuregui 2012). The emerging class of intellectuals in the affluent city of São Paulo was striving to build a new national identity, by dealing with internal and external alterity, both real or constructed. The relationship with alterity was a constant in the history of the country, built upon the radical alterity of the coloniser–colonised–slave triadic relationship that still cannot be reduced to a unity still. The narrative of the triadic identity of Brazil was built around the anthropological myth of the white Portuguese colonisers, the native Indios and the African slaves that managed to build an integrated autochthonous culture (Silva Pereira Jezzini 2010).

The *Anthropophagic Manifesto* claimed that the originality and the creativity of Brazilian modernist culture lay in its ability to elaborate in peculiar ways the European, Indio and African heritage. De Andrade was trying to develop a “modern” way of dealing with the constitutive alterity of Brazilian identity, in which the *Alter* was embodied in the *Self*. The European colonisers who claimed ownership over land and resources, were the *Alter* for the native Indios. At the same time, the population of African descendants, who were considered alien for both the European and Indios, made great contributions to the construction of the Brazilian culture as autonomous from its European roots. Finally, the emerging Brazilian ruling class was striving to emancipate itself from the burdensome legacy of nineteenth century European culture. Each one of the three “races” was doomed to identify with radical alterity in an endless game of mirrors that the Modernist movement was trying to make productive and emancipatory, by exposing the colonial legacy through “the deconstruction of the images of the colonizing power” (de Andrade Tosta 2011, p. 220).

## Anthropophagy and alterity

De Andrade chose the image of the cannibal as the leading metaphor that exposed and ridiculed the colonialist attitude of the Europeans towards the natives. The appropriation of the anthropophagic metaphor also represented an identity construction that reversed one of the major *topoi* that colonisers used to justify the inferiority of the indigenous cultures. However, in the Manifesto, cannibalism was not only about anti-colonial resistance and antagonism towards the European colonising culture. For Brazilian modernism, the metaphor of anthropophagy also epitomised the trope of self-recognition, identity formation and empowerment.

“Down with the reversible world, and against objectified ideas. Cadaverized. The stop of thought that is dynamic. The individual as victim of the system. Source of classical injustices. Of romantic injustices. And the forgetting of inner conquests”. (de Andrade 1991 [1928], p. 40)

The notion of cultural cannibalism itself, if interpreted as appropriation of dominant and hegemonic cultures by subordinated aboriginal groups, reveals a post-colonial impetus, in that it proposes turnings of power relations that give emphasis to the agency of cultural change (de Andrade Tosta 2011).

“Carnal at first, this instinct becomes elective, and creates friendship. When it is affective, it creates love. When it is speculative, it creates science. It takes detours and moves around”. (de Andrade 1991 [1928], p. 43)

The concept of the “cannibal” is entrenched within the very idea of radical Alterity: it first appeared in Columbus’ navigation diary dated 23 November 1492 (Hulme 1986). According to Hulme (ibid.) its etymology is uncertain and could even include the idea of soldiers of the Gran Khan mentioned by Marco Polo. In other words, Hulme (ibid.) maintains that the concept of cannibalism entered into the European languages charged with complex meanings of savagery, radical alterity, orientalism, state of nature, bravery and aggression.

This value persisted over centuries, until the Modernist movements in Europe discovered the power of the cannibalism metaphor through Freud’s *Totem and Taboo *(1919), especially its ability to evoke “savagery” as resistance to the social order, assimilation of the diverse and transformation into a new paradigm (see for instance Francis Picabia’s *Manifeste Cannibale Dada*-1920 DID ANDRADE BUILD ON PICABIA?, Filippo Tommaso Marinetti’s short story *Consigli a una Signora Scettica *(1922), Pasolini’s movie *Porcile *(1969) and Jean-Luc Godard’s film *Week-end *(1979), or the *Tropicalist Movement* in Brazilian music during the 1960s) (Gilebbi 2015).

## The *Alter* in the *Ego*

The case of cannibalism illuminates the way in which colonising cultures built the Ego-Alter relationship through an inclusive exclusion, or a divisive incorporation. In other words, an act of cannibalism that alienates instead of fuses: “In fact, Westerners have no genuine interest in non-Western cultures; they just utilise non-Westerners as the antithetical other for understanding themselves” (Hwang 2015, p. 8). The colonisers’ appetite reduces the *Alter* into a corpse, by physical and symbolic annihilation. The anthropophagic act recognises the *Alter* as an energy, acknowledging and respecting its intrinsic value.

As a mark of Amerindians’ otherness, cannibalism was what the colonising machine manipulated to justify or merely hide some of its acts and intentions (Hulme 1986). The image of cannibal, which came to represent inferiority, allowed conquistadores to exploit indigenous communities, appropriate their knowledge and erase their cultures and societies. The radical *Alter*, defined by opposition, cannot produce valid knowledge. Thus, colonisers do not *learn* from the natives, they *discover* thanks to the advanced Western episteme. *Discovering* conceals acts of appropriation and exploitation, similar to the case of agriculture, where four-sevenths of today’s farming includes plants that were domesticated by Native American botanists.

De Andrade was aware of this historical process and subverted it together with the coloniser–colonised relationship. The act of deglutition itself does not mean to satiate hunger. It is a ritual act of incorporation of the attributes of the “other” (external), overcoming the boundaries of the “Self” (internal) through the assimilation and the expansion of the qualities of the “enemy” (de Andrade Tosta 2011). The *Manifesto* proposed the appropriation of dominant and hegemonic culture by subordinated marginal groups. Consequently, a reformulation of power relations and indigenous agency for cultural change was promoted.

“Cultural cannibalism has a connotation beyond the concepts of borrowing, acquisition, translation. The image of violence, which it embodies—and, in de Andrade’s work, simultaneously subverts because it exposes the colonizer’s brutality—makes it an act of possession (or re-possession), which inverts fallacious views of civilization and savagery, superiority and inferiority, and originality and imitation, thus empowering the peripheral post-colonial” (ibid., p. 218).

The cannibalistic ritual is a transmutation of perspectives, in which the act of incorporation of the other produces the constitution of the Self through the Other (Viveiros de Castro 2015), as opposed to an extractivist consumption of resources in the colonial-capitalist fancy, in which the Other is annihilated.

According to this perspective, the act of cannibalising expresses profound respect and love for the Other. It may seem a paradoxical statement, but we do not incorporate what we consider impure, toxic, dirty or unpalatable. Eating something is the recognition of its intrinsic value. However, the act of eating is also transformational. It implies a preparation (more or less ritualised processing); a social activity (the meal ritual); and digestion (an inner selective transformation). The act of cannibalism is sometimes juxtaposed to the process of learning (Rezende 1929):

“They will have to learn to eat the masters in spicy sauce, which is the best nourishment for a schoolboy and the clearest sign that he starts to become a master himself”. (Pasquali 1948)

De Andrade’s *Anthropophagic Manifesto *was a way of re-structuring the hierarchical relationships between colonisers and the colonised. It was an acknowledgment of autonomy from an oppressing past. It allowed individuals to claim agency and emancipation from family despotism. Finally, the *Manifesto* has also been considered as one of the first statements of a decolonising movement.

“We want the Carib Revolution. Greater than the French Revolution. The unification of all productive revolts for the progress of humanity. Without us, Europe wouldn’t even have its meager declaration of the rights of man”. (de Andrade 1991 [1928], p. 39)

De Andrade acknowledged the role of social sciences in the process of colonial subordination and exploitation:

“Down with all the importers of canned consciousness. The palpable existence of life. And the pre-logical mentality for Mr. Lévy-Bruhl to study”. (ibid.)

Social sciences are one of the agents perpetuating the epistemic stance and the naturalisation of the colonial oppressive system of knowledge.

“If the epistemic conditions of social life were colonised, would not that infection also reach the grammatical level, the very grounds of knowledge? Put differently, couldn’t there also be colonisation at the methodological level? If so, then, any presumed method, especially from a subject living within a colonised framework, could generate continued colonisation. To evaluate method, the best ‘method’ is the suspension of method. This paradox leads to a demand for radical anti-colonial critique”. (Gordon 2014, p. 85)

The anthropophagic metaphor ironically exposed the subject-object hierarchical relationship that, once naturalised, crystallises an unjust epistemic relationship. In the psychological sciences, this also often resulted in the over production of “problem people” (ibid.) through the pathologisation of education, mental well-being, and diversity (Annamma 2017; do Vale Zucoloto, dos Santos and Dazzani 2018).

## Colonisers’ social sciences

The colonising epistemic relationship is the source of a permanent epistemic injustice (Bhargava 2013). The epistemic perspective developed in Europe and North America over the centuries has posed as a universal foundational experience for the production of real knowledge (de Sousa Santos 2018). Its supremacy has been justified, almost as an epistemic Darwinism, and has been considered as the only perspective that is capable of producing significant technological and economic advancements (Hall 2017). This idea of evidence-based progress “granted itself the prerogative of proclaiming its universal validity” (ibid., p. 39) and of relegating alternative epistemologies into “nonexistence, radical invisibility, and irrelevance” (ibid., p. 25). According to de Sousa Santos, the primacy has actually been a self-fulfilling prophecy obtained through “overriding economic and military power” (ibid., p. 39). It is not the universal validity that granted epistemic primacy to a part of the world, rather its violent action that imposed a monological epistemic perspective as the only valid perspective. It is important to note that this is not a discourse to diminish the validity of scientific work or to overlook the production of knowledge aided by scientists. Replacing a monological and hegemonic discourse with another form of epistemic monoculture would result in the reproduction of the same oppressive system.

Monological epistemology is produced and reproduced through the power of legitimatising *who* can research *what*. It defines a unilateral epistemic relationship in which only one can be the knower, and the object cannot really inquire in return (ibid.). In cross-cultural research, for instance, the epistemological relationship implies that one dominant culture is compared with a non-dominant one, embedding a specific perspective and using theoretical categories that have emerged from the dominant culture. One can seldom read a cross-cultural study, for instance, between North American and Ethiopian adolescents from the epistemological perspective of Ethiopian conceptual categories. The colonialist–capitalist gaze is always monodirectional, as the observer and observed are not interchangeable roles and the microscope is always held by the dominant culture. According to Gordon (2014), this means that the observed tends to be constructed as inherently problematic. The naturalisation of specific categories of people as objects of study—often coinciding with marginalised categories such as migrants, disabled, etc.—leads to the construction of “problem people”.

“In the human sciences, the problem becomes particularly acute in the study of problem people. Such people also misbehave in disciplinary terms. The failure to squeeze them into disciplinary dictates, from a disciplinarily decadent perspective, is proof of a problem with the people instead of the discipline. It serves as further proof of the pathological nature of such people”. (Gordon 2014, p. 87)

It is rare to read a study about the welfare system from the perspective of migrants or in which the researchers are the very migrants. This unidirectionality of the gaze leads to a unilateral selective appropriation by the dominant groups. For example, psychology selectively appropriates some isolated concepts such as mindfulness from a whole philosophical and psychological system of knowledge, which is then made invisible and relegated to the role of the object rather than the producer of knowledge.

Combined with the capitalistic idea of scientific competitiveness, unidirectionality of gaze also leads to selective promotion or the inhibition of ideas, because the dominant epistemology decides not only about who is legitimatised to produce knowledge, but also what topics are worthy of interest, through the competitive system of funding, for instance. A research topic is considered relevant only from the perspective of a colonising-capitalistic value of use. The people or the communities who are the objects of study are never invited to peer-review the funding application or to decide what projects are worth funding. Competitive funding negotiations and evaluations take place at the level of political and scientific establishments, with the only requirement being enforced that of including organisations that represent the final “beneficiaries”, such as NGOs or trade unions. If one presents a funding application for a research project on a rural community, for instance, the application is often peer-reviewed solely by scientists and rarely by members of the community itself. It would be interesting to observe the relationship between the rejection rate of proposals (which funding agencies claim to be very high as point of merit for the quality of competition) and the type of projects that are funded. It is often claimed that science should be free from any political influence, including public opinion. But the capitalistic competitive system of research funding itself deeply politically influences science inside and outside academia (Barnes 2019). Psychology is still reluctant to conduct research *with* (rather than *about*) communities, to be held accountable, and to be truly participative in the construction of knowledge (Normann 2021).

The monological and unidirectional construction of epistemic relationships finally leads to an exporting/exploiting dynamics of theoretical constructs, or the so-called mainstream. A stream of theoretical constructs is exported from some epistemic centre of production to the rest of the world. The opposite stream consists of the importing of “data” that converts the epistemic marginal areas into territory to exploit for the “mining” of data.

### Epistemic monoculture

The colonialist perspective works by over-imposing its universalised interpretive categories across different local realities. The decolonising approach, on the other hand, exposes the Western Euro-North American perspective not as a universal one but rather as “an indigenous psychology created out of a set of local cultural assumptions and values about the Western, individual self” (Bhatia 2018, p. xxiii).

**Fig. 2:**
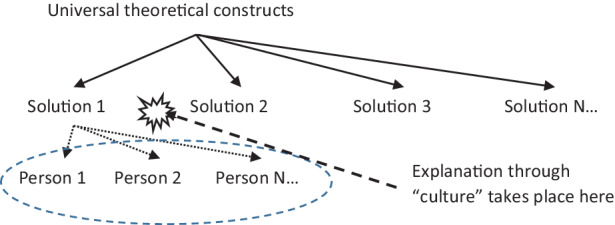
Current cross-cultural approach

The current cross-cultural approach (Fig. 2) consists of collecting inductive evidence about personal variations of a psychological and cultural (read national) pattern, in conjunction with a pre-existing hypothetic construct that is usually developed in a Western tradition of two or more different local realities.

Therefore, variability (or similarity) in a limited number of binary dimensions (e.g. dependence/independence, collectivistic/individualistic, attachment styles, etc.) is explained by the belonging to the “culture”, which becomes an independent variable. This generalisation is produced through the inductive accumulation of instances and is validated by the assumed universality of the construct itself. Combined with the Occam’s razor assumption, which is controversial when it comes to such complex phenomena as ecosystem relationships (Riesch 2010), this leads to a point in which a final leap to attribution is required: causal explanation is attributed either to culture or to individual traits.

This summarises the main critique from decolonising perspectives (Bhatia 2018): psychological constructs are not to be assumed as universal, they are the product of an indigenous, historically situated scientific tradition that has mutually fed into the colonialist process. Therefore, we need new ways of thinking about the idea of universality itself, not as an attribute of a single construct.

## Epistemological deserts

The anthropophagic metaphor in this case would constitute a rejection of epistemic *food deserts* (Whelan, Wrigley, Warm and Cannings 2002), where some areas of knowledge production and scholars are forced to consume processed and imported knowledge constructs, thereby reducing the diversity of knowledge itself. The capitalist agricultural and food processing business extracts raw materials and establishes extensive monocultures in the global South or lower income countries. Raw materials are then processed, marketed, and exported by multinational companies to be sold back to the countries of origin at higher costs. Similarly, data are mined from groups who are the objects of study, processed in the mainstream centres of knowledge production, only to be sold back to the periphery by fashionable editorial multinational companies. The anthropophagic metaphor is not about eating the food processed by the coloniser, but eating the coloniser themselves, acknowledging their capability of producing sophisticated knowledge, but also reclaiming agency of digesting and producing new forms of knowledge according to the principle of diversity. *Diversity* is most certainly the key to any ecological system for life to thrive (de Sousa Santos 2018). In the next section of this paper, we discuss how the anthropophagic metaphor can help to imagine a psychological science that has the ability to promote *psychodiversity*.

## A global psychology

The psychological sciences experience an epistemological conundrum when dealing with diversity, instability, development, and variability (Valsiner 1984). Prevailing statistical approaches tend to minimise variability in order to attain generalisability and replicability of results. Unexplained variance is often ignored because of the assumption that it possesses minimal relevance to what is being studied. Lewin (1931) refers to this as the *typological mode of thinking* in psychology, which “extracts the moment of stability from the psychological phenomena and attempts to explain these phenomena in their ideal (average or prototypical) cases” (Valsiner 1984, p. 468). The opposite, which is a more dynamic perspective, is the *variational mode of thinking*, which:

“takes the whole range of variability within the phenomena into account and tries to explain that variability as it constitutes a lawful phenomenon. The two modes are approaching psychological phenomena from opposite perspectives—what is ‘error’ or ‘irregularity’ for the first constitutes the target phenomenon for the second, and vice versa” (ibid.).

The variational mode of thinking is thus able to grasp “complex, fluid, and self-transforming phenomena”. (Tateo and Marsico 2021, p. 2). Life favours the systematic amplification of variability and hierarchical differentiation (Maruyama 1963). Therefore, it is difficult to understand the complexity of a living ecosystem by focusing on a narrow range of non-variational features. An ecosystem where variability is reduced, such as huge industrial monoculture, would only be economically efficient from the perspective of capitalism. However, from an environmental perspective, it would be extremely vulnerable and not resilient.

We must then acknowledge variability, diversity and differentiation as positive features that are necessary for the development of living systems (Valsiner 2005), that proceed “from a state of relative lack of differentiation to a state of increasing differentiation, articulation, and hierarchic integration” (Werner and Kaplan 1956, p. 866). However, differentiation and specialisation *within* systematic organisation does not mean competition, rather it encourages the establishment of hierarchical integration and new part-whole relations. The capitalistic and colonialist perspective, which is based on the principle of competition between monocultures (or epistemological monocultures) opposes this. The current global order of knowledge is seemingly based on a centre–periphery typology, where the centre is located in geopolitically dominant areas. This is a spatial static organisation concept; and not an ecological and developmental one. Monoculture and purity are toxic in all systemic organisations. A living, dynamic psychological science should therefore orient itself to what de Sousa Santos (2018) called *ecologies of knowledge*: different types of knowledge should fertilise each other and form a complex epistemological ecosystem, where our understanding of the human being, viewed as a whole (complex organism) and part of a whole (complex ecosystem), can thrive.

A particularly pernicious form of capitalistic epistemic monoculture in psychology is the cultivation of intra- and interdisciplinary boundaries. Such an “inward path of disciplinary solitude” leads to what Gordon (2014) calls “disciplinary decadence” (p. 86).

“This is the phenomenon of turning away from living thought, which engages reality and recognises its own limitations, to a deontologised or absolute conception of disciplinary life. The discipline becomes, in solipsistic fashion, the world. And in that world, the main concern is the proper administering of its rules, regulations, or, as Fanon argued, (self-devouring) methods. Becoming ‘right’ is simply a matter of applying, as fetish, the method correctly. This is a form of decadence because of the set of considerations that fall to the wayside as the discipline turns into itself and eventually implodes”. (Gordon 2014, p. 86)

Additionally, psychological training promotes a hyper-specialised technical training that impoverishes the sensitivity and intellectual curiosity of the students, and overlooks the importance of a humanistic education (Tateo and Marsico 2021).

“Decay, although a natural process over the course of time for living things, takes on a paradoxical quality in disciplinary formation. A discipline, e.g., could be in decay through a failure to realise that decay is possible. Like empires, the presumption is that the discipline must outlive all, including its own purpose. In more concrete terms, disciplinary decadence takes the form of one discipline assessing all other disciplines from its supposedly complete standpoint. It is the literary scholar who criticises work in other disciplines as not literary. It is the sociologist who rejects other disciplines as not sociological”. (Gordon 2014, p. 86)

The response to disciplinary decadence, which refers to the way of preserving psychology from the exhaustion of a monological epistemology, is usually the appeal of interdisciplinarity. Interdisciplinarity is eventually the “other side” of disciplinary boundaries:

“This is because presumed disciplinary completeness of each discipline is compatible with disciplinary decadence. Disciplines could simply work alongside each other like ships passing in the night. A more hopeful route is transdisciplinarity, where disciplines work through each other; yet although more promising, such a route is still susceptible to decadence so long as it fails to bring reality into focus. But doing that raises questions of purpose. It raises considerations that may need to be addressed in spite of disciplinary dictates. I call this process a teleological suspension of disciplinarity. By that, I mean the willingness to go beyond disciplines in the production of knowledge. This ‘beyond’ is, however, paradoxical. In some instances, it revitalizes an existing discipline. In others, it generates a new one”. (ibid., p. 87)

To counter epistemological monoculture, we advocate a psychology that cultivates diversity and transdisciplinary crossing, rather than a hyper-fragmented technical form of knowledge. The *Anthropophagic Manifesto* claims the irreducibility of an ecological system of knowledge (de Sousa Santos 2018) to a single monoculture dominated by a colonialist–capitalist epistemic order.

Anthropophagy in psychology is a metaphor for an open systemic organisation and ecological exchange. Living ecosystems cultivate *biodiversity, anthropodiversity* and *psychodiversity*, which cannot be reduced to a common type of specimen with differences in magnitude, like for instance applying the same scale after a cultural adaptation or developing culture-free instruments. Psychological sciences should strive to learn from transdisciplinary areas of knowledge such as ethnobiology (Albuquerque and Alves 2016), where the objects of study consist of unique configurations of local ecosystems, including both human and non-human organisms. Ecosystems cannot be transferred or replicated, nor can they be reduced to monocultures. Nevertheless, one can produce universal knowledge of unique historical developmental processes (Valsiner 1984).

We propose an ecological perspective that considers local realities as complex ecosystems, where the unique relationships between sub-parts are not replicated elsewhere. Culture is the whole complex of solutions that humans have developed over time to solve universal existential problems. Therefore, it is difficult to understand and generalise the relationship between humans and their world simply by comparing results (typological mode of thinking in cross-cultural psychology). Psychology should also not limit itself to an individual centric analysis (Rodax and Benetka 2021). By generalising outcomes, it becomes easy to trivially conclude that every person and every culture has developed similarities or differences in dealing with existential problems (e.g. educating offspring, regulating conflict, reproduction etc.). By design, the colonialist–capitalist order will compare results against a dominant metric, leading to the emergence of the abyssal line of division between “developed” and “undeveloped” people (de Sousa Santos 2018).

**Fig. 3:**
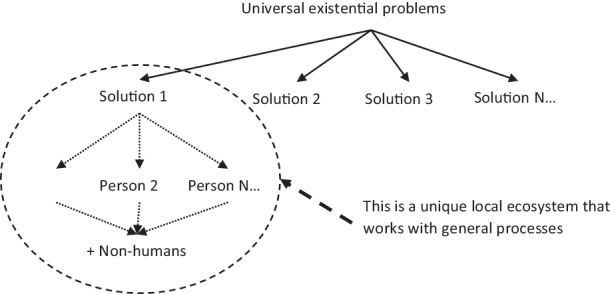
Ecosystemic approach to cultural differences

From ethnobiology, one can learn the uniqueness of ecological outcomes: it makes no sense to compare, for instance, a banana tree in Brazil with an apple tree in Norway, on a hypothetical construct of “treeness” and then attribute this difference to the variable of “cultivation”. One cannot generalise the outcomes, but general laws of systemic organisation can be generalised, which is the case in the variational mode of thinking in psychology (Fig. 3).

Knowledge about the unique functioning of local ecosystems can produce generalisable knowledge about the process dynamics in ecological human systems that could become an interpretative tool for different local realities. For instance, recently, countries have begun to evaluate all their school systems against the same standard in order to develop “efficient” systems of resource allocation. This leads to the false belief that teaching/learning processes are universal. If the comparison is made between a sufficient number of students, then the personal traits can be excluded as a cause and the most influencing factor becomes the school system itself. From the perspective of the dominant colonial–capitalistic logic that informs the methodology itself, the logical conclusion would be that the best system is the one that is able to perfectly comply with an educational monoculture. Therefore, all the other school systems should also be adequate.

An ecosystemic approach, such as the one that cultural psychology is aimed at developing, would instead consider the universal existential problem of educating children as a starting point and then understand different local configurations and the history of their unique development under local ecological conditions. First, this would invalidate the assumption of assessing the systems by comparing the outcomes: non-sense of comparing a palm and an apple tree by their fruits on the same standard and then claiming that every country should cultivate apples. Then, one could formulate laws of systemic organisation, such as the concept that schooling is a solution to the problem of educating the community offspring according to the local system of values, which provides an image of how the child should strive to become (but also what they should not become).

## Conclusion: a manifesto for global psychology?

Can one imagine an epistemology of psychology that is able to incorporate the ecosystemic principles we have discussed above? Can one eventually draft an *Anthropophagic Manifesto of Psychology*?

- Anthropophagy in psychology is a metaphor for unique open-systemic developing organisations towards increasing differentiation, articulation and hierarchic integration.
- Differentiation and specialisation *within* systemic organisation is not competition, rather it is the establishment of intransitive hierarchy and new part-whole relations.
- Anthropophagy is not an act of oppression/silencing; instead, it is the honest recognition of the value of alterity through ingestion and digestion of the enemy. Acknowledging the value of the enemy contributes to strengthening of one’s own identity.
- In this sense, there is no epistemic “enemy”, rather epistemic differentiation that enriches the cognitive ecosystem.
- Centre-periphery is a spatial and static organisation concept, whereas part-whole is a systemic dynamic concept.
- Purity is toxic in all living systemic organisations: living systems cultivate *biodiversity, anthropodiversity, psychodiversity*. *Pure* substances are toxic to living systems: they always exist as compounds.
- Therefore, to oppose colonialist, nationalist and neoliberalist competition in knowledge making, and to oppose academic fences, the solution is to eat masters in spicy sauce!

All these axioms lead the science of psychology to the question of how can one study complex psychological issues. Contemporary cultural psychologies are perhaps at the frontlines, engaging in efforts to address this issue (Valsiner et al. 2016; Marsico and Valsiner 2018) by endorsing the notion of variability in its fascination with phenomena while proceeding along the lines of generalisable psychological processes.

Despite taking different forms, cultural psychologies are unified as being a part of science. In its ideal form, science has no national boundaries. There is no separate “American psychology”, “Australian psychology”, “Russian psychology”, “indigenous psychology” etc., but one general science that benefits from the work of scientists in any country. This restoration of the international nature of knowledge creation will bring psychology back from having become a social tool employed to assert one country’s dominance over another, to a universal domain of knowing (*Wissenschaft*).
